# Pan filarial-nested PCR with gelified tubes: a new approach towards point-of-care testing for human filariasis

**DOI:** 10.1590/0074-02760250337

**Published:** 2026-09-25

**Authors:** Raquel Capote-Morales, Agustín Benito, Pedro Berzosa, Vicenta González, Luz García, Alba Sastre-González, Akeem Abiodun Akindele, Olusola Ojurongbe, Thuy-Huong Ta-Tang

**Affiliations:** 1 Instituto de Salud Carlos III, National Centre of Tropical Medicine, Laboratory for Scientific-Technical Cooperation in Tropical Diseases, Madrid, Spain; 2 Complutense University of Madrid, Faculty of Biological Sciences, Madrid, Spain; 3 Biomedical Research Networking Centre of Infectious Diseases, Madrid, Spain; 4 Alcalá University of Madrid, Faculty of Sciences, Alcalá de Henares, Spain; 5 Ladoke Akintola University of Technology, Medical Laboratory Science Department, Ogbomoso, Nigeria; 6 Ladoke Akintola University of Technology, HRH-Centre for Emerging and Re-Emerging Infectious Diseases, Ogbomoso, Nigeria

**Keywords:** nested PCR, gelified tubes, point-of-care test, human filariasis, Loa loa, Mansonella perstans

## Abstract

**BACKGROUND:**

In recent years, traditional point-of-care tests (POCTs) for diagnosing human filariasis have demonstrated substantial limitations, raising concerns regarding their reliability in the current epidemiological context, in which numerous countries are approaching the elimination of human filariasis. Consequently, there is a need for new molecular tests that allow the simultaneous and reliable detection of multiple human filarial species, even in cases of very low microfilaremia.

**OBJECTIVES:**

The objective of this study was to convert the *Fn-PCR* from its original manual format to the new *gelified tube-filarial-nested polymerase chain reaction* (*Gel-Fn-PCR*), which overcomes some of the disadvantages of traditional molecular techniques thanks to its gelified tube format.

**METHODS:**

A total of 661 dried blood spots, along with their corresponding stained blood smears, were employed for the optimisation and validation processes. The results of the *Gel-Fn-PCR* were compared to the microscopic diagnosis.

**FINDINGS:**

The *Gel-Fn-PCR* demonstrated good sensitivity and specificity for single-species infections (91.6% and 83.0% for *Loa loa*, and 95.0% and 97.7% for *Mansonella perstans*, respectively). However, for mixed infections, the sensitivity was low (43.9%), while specificity remained high (99.0%).

**MAIN CONCLUSIONS:**

The assay proved to be effective for diagnosing single-species infections, even in cases with very low microfilaremia; however, its performance in identifying mixed infections was reduced, particularly when one species was present in much higher abundance than the other. Although not yet feasible as a fully deployable POCT, this study demonstrates the potential of integrating gelification with PCR for human filariasis diagnosis.

Diagnostic tools are essential in the management and control of neglected tropical diseases (NTDs). Accurate and effective diagnostic testing facilitates the timely identification of cases and treatment, as well as critical activities such as disease mapping, population screening, and epidemiological surveillance.^1^ These efforts are vital for accelerating progress toward the elimination of NTDs.

Currently, the commercially available point-of-care tests (POCTs) are immunoenzymatic tests that detect either circulating antibodies, such as Brugia Rapid Test (Reszon Diagnostics International Sdn. Bhd., Selangor, Malaysia) for detecting antibodies specific to *Brugia malayi* and/or *B. timori*, and Bioline^TM^ Onchocerciasis IgG_4_ rapid diagnostic test (Abbot, Lake Forest, IL, USA) for detecting IgG_4_ antibodies to *Onchocerca volvulus* Ov16 antigen, or circulating antigens, like the BinaxNOW Filariasis test kit (Abbott, Chicago, IL, USA), Bioline^TM^ Filariasis Test Strip (FTS) (Abbott, Chicago, IL, USA), and STANDARD Q Filariasis Antigen Test (QFAT) (SD Biosensor, Suwon, Republic of Korea) for the qualitative detection of circulating filarial antigen from *Wuchereria bancrofti*.^2^ ^,^ ^3^ ^,^ ^4^ ^,^ ^5^ ^,^ ^6^ However, immunoenzymatic tests have notable limitations that reduce their effectiveness for monitoring ongoing transmission or assessing treatment efficacy in elimination programs. Antibody-based tests, like the Brugia Rapid Test or the Bioline^TM^ Onchocerciasis, may generate false-positive results due to circulating antibodies that remain after treatment, therefore, not being able to distinguish between active and past infections. Antibody-based tests detect the host immune response rather than the presence of the parasite itself. Positive serology does not necessarily indicate active infection, because circulating antibodies can persist for long periods after successful treatment or parasite clearance. Another limitation of the Bioline^TM^ Onchocerciasis is that, according to the study published by Gbakima et al. in 1996, the immunoglobulin G and E reactivity during *O. volvulus* infection in children from two to 15 years old, approximately 20% of individuals do not generate an antibody response against the Ov16 antigen, which causes false-negative results.^7^ ^,^ ^8^ Antigen-based tests can also generate false-negative results due to either low or high antigen levels.^9^ The BinaxNOW Filariasis test ki^t®^ has a three-month shelf-life, has to be cold-stored, and its results may vary over time.^4^ ^,^ ^7^ Cross-reactivity with *L. loa* has also been reported for both the BinaxNOW Filariasis test kit^t®^ and Bioline^TM^ Filariasis Test Strip tests.^10^

Nucleic acid-based tests, which target parasite DNA, offer greater sensitivity and specificity than immunoenzymatic tests. However, molecular approaches are often deemed unsuitable for POCT settings due to the high cost of reagents, the need for specialised equipment, multiple procedural steps, and the increased risk of contamination.^9^ ^,^ ^11^ In 2010, Ta-Tang et al. developed a highly sensitive and specific nested polymerase chain reaction for the diagnosis of filariasis, capable of detecting all human-infecting filarial species simultaneously, known as *filaria-nested PCR* (*Fn-PCR*).^12^ However, it faced the aforementioned usual problems, as well as others encountered when trying to implement the technique in laboratories located in endemic countries, such as the cost of transportation and the difficulty of keeping the reagents at an optimal temperature both during transportation and once stored at the destination.

To overcome these limitations, the process of gelification, developed by Biotools Biotechnological & Medical Laboratories S.A. (Madrid, Spain) in 2001, was taken into consideration.^13^ ^,^ ^14^ This process enables the incorporation of all necessary PCR reagents, except water and isolated DNA, into a gel matrix within the PCR tube. The reagents remain stable, with no significant loss of enzymatic activity, for up to one month at room temperature and over six months at 4ºC.^14^ Gelification is a more cost-effective alternative to traditional preservation techniques due to its complete automation and the capacity to process multiples of 96 tubes simultaneously.

Integrating a nucleic acid-based test with the gelification technique would align with the World Health Organization's roadmap for NTDs through 2030, which highlights the urgent need for the development of more sensitive, specific, non-invasive, cost-effective, and easy-to-implement diagnostic methods, particularly those integrated into multiplex platforms.^1^ As onchocerciasis and lymphatic filariasis microfilaremia has significantly declined in several endemic regions due to the great effectiveness of the mass drug administration campaigns implemented through control and elimination programs, developing new feasible molecular methods has become essential for the diagnosis of human filariasis.^15^-^21^

Therefore, the objective of this study was to convert the original manual *Fn-PCR* into a gelified tube format, termed *gelified tube-filarial-nested PCR* (*Gel-Fn-PCR*) by applying the gelification process to the reaction components. This modification was intended to simplify the nested PCR workflow by reducing manual preparation steps and handling, thereby facilitating assay implementation in routine laboratory settings. In addition, the gelified format aims to improve standardisation, reduce the risk of contamination associated with multiple reaction preparations, and lower operational costs. Finally, the *Gel-Fn-PCR* was optimised and validated for the laboratory diagnosis of human filariasis.

## MATERIALS AND METHODS

*Microscopy* - A total of 661 blood smears from Equatorial Guinea and Nigeria were analysed. The microscopic analysis of the thick blood smears from Equatorial Guinea was performed by researchers of the National Centre of Tropical Medicine (CNMT, Spain), while the microscopy of Nigerian samples was conducted by microscopists of Ladoke Akintola University of Technology (LAUTECH, Nigeria). Equatorial Guinea thick blood smears were obtained via finger-prick and stained with Giemsa (3%), whereas for Nigerian blood smears, venous blood was obtained from each patient, and initially, blood was directly examined for the presence of microfilariae using a wet blood film. Those filarial-positive samples, from 5 to 10 µL of blood were taken to prepare thin blood smears, fixed in methanol and stained with haematoxylin. Both in the CNMT and LAUTECH, all stained blood smears were examined by two different expert microscopists. Each thick blood smear contained approximately 20 µL. The entire smear was systematically screened and the number of microfilariae was recorded. Microfilarial density was subsequently calculated and expressed as microfilariae per millilitre of blood (mF/mL) according to the volume of blood examined. The detection of the microfilariae was performed under the 10x and 40x objectives, whereas identification of filarial species was achieved under the 100x oil-immersion objective to visualise the main morphological characteristics of the microfilariae.

*Sample collection and preparation of dried blood spots (DBS)* - To optimise and validate the *Gel-Fn-PCR* technique, a total of 661 DBS were used. The DBS samples belonged to the repository of CNMT (collection number C.0005278/ISCIII/Spain, Instituto de Salud Carlos III, in accordance with Spanish Law RD 1716/2011. Article 22.1). The samples from Nigeria were collected in 2015. The DBS samples were prepared impregnating the Whatman paper with the venous blood. Samples from Equatorial Guinea were collected in 2019, and the DBS samples were prepared by impregnating the Whatman paper with blood obtained via finger-prick.

*DNA isolation* - DNA isolation was performed using the Investigator STR GO! Lysis Buffer^®^ (QIAGEN, Germany). A detailed description of the procedure was published in Capote-Morales et al.^22^ The advantage of this DNA isolation method is the requirement of only basic and inexpensive laboratory equipment, such as a vortex mixer, a thermal block, and a centrifuge.

The use of DBS samples also simplifies the sampling process by requiring a small sample volume and being minimally invasive. Given that DBS samples remain stable for long periods of time at room temperature, their use reduces transportation costs and does not require special storage conditions.^23^ ^,^ ^24^ ^,^ ^25^

The combination of DBS samples with this DNA isolation method makes the process rapid and easy to perform, making it particularly suitable for implementation alongside the *Gel-Fn-PCR* in low-income settings.

*Gel-Fn-PCR: optimisation* - The *Gel-Fn-PCR*, adapted from the *Fn-PCR* developed by Ta-Tang et al. in 2010, targets partially the 18S (small subunit ribosomal RNA), ITS1 (first internal transcribed spacer), and partially the 5.8S regions for filarial detection and identification.^12^

The original *Fn-PCR* had a 50 µL final reaction volume, but the *Gel-Fn-PCR* was reformulated to a final reaction volume of 25 µL, according to the gelification process. The isolated DNA from positive and negative filarial samples was tested both pure and diluted with distilled water ten times. Likewise, the first PCR product to be incorporated into the second PCR was tested both non-diluted and diluted ten times in distilled water.

The optimisation process with DNA polymerase (Biotools, B & M Labs, S.A., Madrid, Spain) and primers was carried out by Biotools, preparing a series of formulas containing different concentrations, which were blindly checked in the laboratory to determine which one yielded the best results. Two different DNA polymerase enzymes from Biotools were evaluated to minimise non-specific amplification.

In addition, the FIL2F primer (GGTGAACCTGCGGAAGGATC) from the second PCR was replaced by the ITS1F-BIS primer (5′-GGTGAACCTGCRGMWGGATC-3′), a degenerate version of the original FIL-2F primer, was used in this study to improve amplification of genetically diverse *L. loa* isolates.

For each reaction performed, appropriate positive and negative controls were always included. Positive controls were DBS samples of *L. loa* and *M. perstans* diagnosed by microscopy, as well as by other validated molecular diagnostic methods available in our laboratory; negative controls were DBS samples of filarial microscopy-negative individuals from filarial endemic regions that were negative for filarial infection but could be positive or negative for other tropical parasites, such as *Plasmodium* spp. All samples were run in duplicate. If the duplicates varied (one positive, one negative), the samples were rerun in triplicate.

During the optimisation process, the gelified tubes were stored at 4ºC. Regarding the PCR amplification program, no modifications were introduced.

In the course of the initial optimisation phase, a subset of samples was analysed in parallel using both the original *Fn-PCR* and the *Gel-Fn-PCR* to confirm the equivalence of the two approaches. Once comparable performance was established, subsequent analyses were performed using only the *Gel-Fn-PCR* assay. For the evaluation of diagnostic performance in the full dataset, microscopy was used as the reference method, as it represents the routine diagnostic standard in the study settings.

*Validation* - To assess the diagnostic performance of the *Gel-Fn-PCR* and to determine its validity as a reliable alternative diagnostic method for filarial infections, the final optimised *Gel-Fn-PCR* was subjected to validation testing on 661 DBS clinical samples in the laboratory. The complete, final workflow of the *Gel-Fn-PCR* assay, encompassing all procedural steps from clinical sampling to result visualisation, is depicted in Fig. 1.

In selected cases with inconclusive or unexpected results, additional confirmatory molecular assays were performed using previously published methods.

**Fig. 1:**
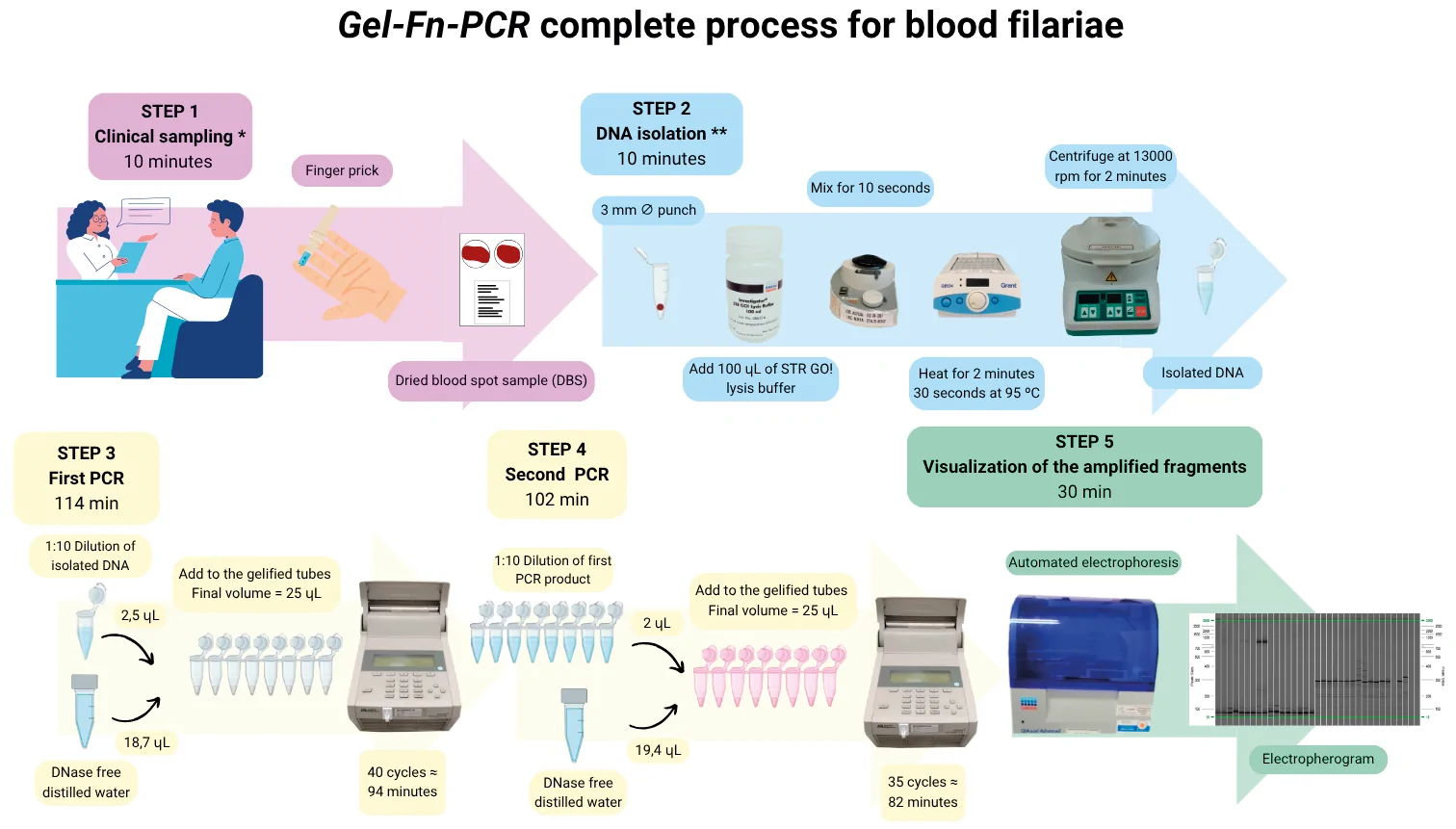
depiction of the *Gelified tube-filarial-nested polymerase chain reaction (Gel-Fn-PCR)* complete process. Workflow of the *Gel-Fn-PCR* assay for the detection of filarial parasites. Asterisks (*) indicate steps that differ when the type of clinical sample are skin snips used for the detection of *Onchocerca volvulus*, a modified workflow is applied compared to the procedure used for blood samples targeting blood-dwelling filarial species (*e.g.*, *Wuchereria bancrofti*, *Loa loa* and *Mansonella perstans*). *In case of onchocerciasis suspicion, two skin snips from the iliac crest should be taken and put into an Eppendorf with 200 µL of physiologic solution. **Skin snips samples must undergo an additional 2 h of incubation with proteinase K at 56ºC before DNA isolation. Once skin snips are completely disrupted, 50 µL of the solution are taken and spotted onto Whatman paper.

*Visualisation of amplified fragments* - Since the *Gel-Fn-PCR* technique is a conventional PCR, amplified DNA fragments were visualised using the Qiaxcel Advanced automated electrophoresis system (QIAGEN, Germany). The identification of the filarial species was determined based on the size of the amplified fragment from the second PCR, and by comparing it with the size of the positive controls included in the PCR reaction.

The Qiaxcel automated electrophoresis system allows rapid, standardised, and high-resolution fragment analysis. However, conventional agarose gel electrophoresis can also be used as an alternative, particularly in resource-limited settings.

*Statistical analysis* - To evaluate the capacity of the *Gel-Fn-PCR* as a diagnostic method, the obtained results were compared with microscopy method, considered as the gold standard for diagnosing human filariasis. Statistical analyses were performed using WinEpi 2.0 software.^26^ The calculated parameters included the sensitivity, specificity, positive predictive value, negative predictive value were calculated based on standard definitions for diagnostic test evaluation, and 95% confidence intervals (95% CI) were computed for each parameter. Agreement between methods was assessed using Cohen's kappa index with corresponding 95% CI. The recommendation was to have a high sensitivity and a high specificity for *Gel-Fn-PCR* method.^27^

*Ethics committee statement* - All clinical samples (DBS and thick blood smears) were authorised by the director of the National Centre of Tropical Medicine for its use in this study, with the unique purpose of optimising and validating the *Gel-Fn-PCR*. Samples were irreversibly anonymised and no additional ethical approval or informed consent was required for the present study.

## RESULTS

*Microscopic diagnosis* - Filarial parasites were identified in 54.61% samples (361/661). Among the positive samples, 55.96% (202/361) were confirmed as mono-infections with *L. loa*, 5.54% (20/361) as mono-infections with *M. perstans*, and 38.50% (139/361) as mixed infections with *L. loa* and *M. perstans* (Fig. 2). A detailed depiction of the results is provided in Table I.

**Fig. 2:**
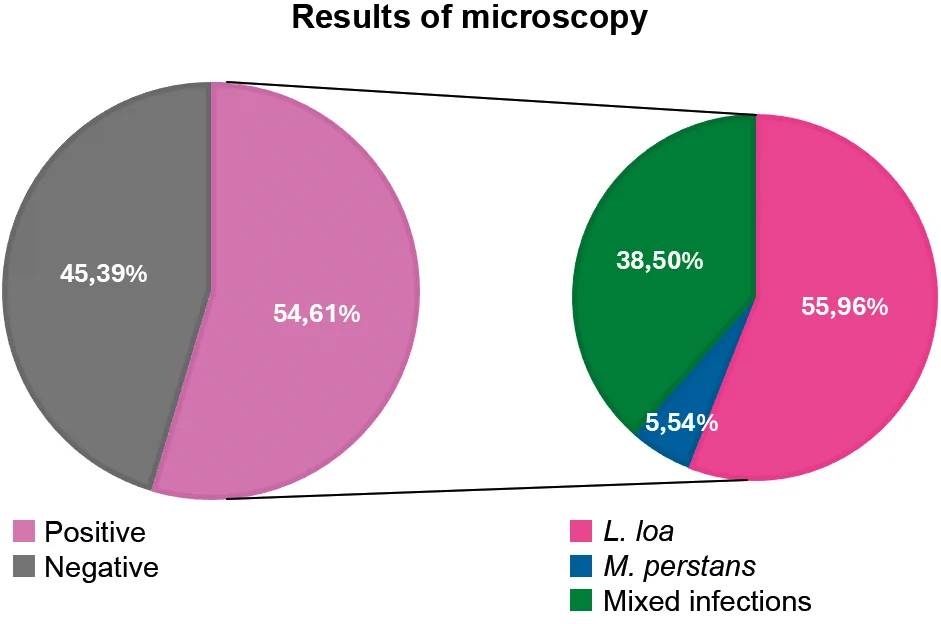
summary of the results of the microscopic diagnosis. Sectoral chart illustrating the proportional distribution of the different infections and filarial species identified through microscopic examination.

**TABLE I:** Comparative results between microscopy and Gelified tube-filarial-nested polymerase chain reaction (Gel-Fn-PCR)

			Microscopy				
			Positives (54.61%, 361)			Negatives (45.39%, 300)	Total
			*L. loa*	*M. perstans*	Mixed infection		
Gel-Fn-PCR	Positives (54.92%, 363/661)	*Loa loa*	**185**	0	62	16	263
		*Mansonella perstans*	0	**19**	14	1	34
		Mixed infection	4	0	**61**	1	66
	Negatives (45.08%, 298/661)		13	1	2	**282**	298
	Total		202	20	139	300	661

Overall agreement between microscopy and *Gel-Fn-PCR* was observed along the diagonal of the matrix (true concordant results). Discordant results were primarily associated with differences in the detection of mixed infections and cases classified as negative by one of the methods.

The median microfilaremia was 2700 mF/mL for *L. loa* in mono-infection, with microfilaremia ranging from 50 to 64850 mF/mL. For *M. perstans* mono-infection, the median microfilaremia was 850 mF/mL, with microfilaremia ranging from 500 to 2450 mF/mL.

Regarding mixed infections, the median microfilaremia was 2950 mF/mL (ranged 50-76700 mF/mL) and 150 mF/mL (ranged 50-5550 mF/mL) for *L. loa* and *M. perstans,* respectively.

*Sample collection and preparation of DBS* - Out of 661 DBS samples, 49.77% (329/661) were collected in Equatorial Guinea, and 50.23% (332/661) were collected in Nigeria. 76.60% (252/329) of Equatorial Guinea samples were positive: 55.16% (139/252) were mixed infections (*L. loa* and *M. perstans*), 36.90% (93/252) were *L. loa* mono-infection, and 7.94% (20/252) were *M. perstans* mono-infection. 32.83% (109/332) of Nigerian samples were positive, all of which were *L. loa* mono-infection.

*Gel-Fn-PCR: final optimised conditions of the Gel-Fn-PCR assay* - The following section describes the final optimised conditions of the *Gel-Fn-PCR* assay established after systematic evaluation of multiple reaction parameters.

The final *Gel-Fn-PCR* contained 1 U/µL of DNA polymerase in both the first and second PCR, 0.5 µM of each FIL1F and UNI1R primers (first PCR), as in the original *Fn-PCR*, 0.05 µM of each ITS1F-BIS and FIL2R primers (second PCR), concentrations higher than the *Fn-PCR,* at a final volume of 25 µL. The volume of isolated DNA diluted 1:10 in distilled water used for the first PCR was 2.5 µL, and 2 µL from the first PCR product diluted 1:10 in distilled water were used for the second PCR.

*Validation* - The *Gel-Fn-PCR* identified 54.92% (363/661) as filarial-positives and 45.08% (298/661) as negative for filarial parasites. Among the positive samples, 72.45% (263/363) samples were identified as *L. loa* infections, 9.37% (34/363) samples as *M. perstans* infections, and 18.18% (66/363) samples as mixed *L. loa* and *M. perstans* infections (Fig. 3). A comparison of the results of *Gel-Fn-PCR* against the reference method, microscopy, is presented in Table I. Overall agreement between microscopy and *Gel-Fn-PCR* was observed mainly along the diagonal (true concordant results), whereas discrepancies were observed in cases classified as mixed infections or negative by one of the methods.

**Fig. 3:**
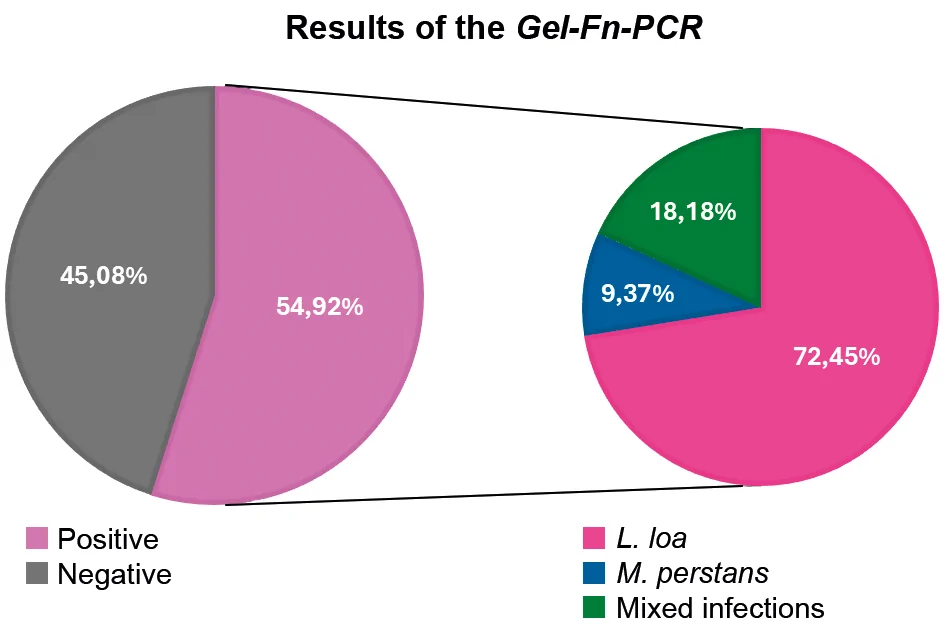
depiction of the *Gelified tube-filarial-nested polymerase chain reaction (Gel-Fn-PCR)* results. Sectoral chart indicating the proportional distribution of different infections and filarial species detected by the *Gel-Fn-PCR* assay.

Microscopic diagnosis was able to detect more mixed infections, 21.03% (139/661), than the *Gel-Fn-PCR*, 9.99% (66/661). Most mixed infections missed by *Gel-Fn-PCR* (76/139) were classified as mono-infection, predominantly as *L. loa* (62/139) and to a lesser extent as *M. perstans* (14/139). To overcome these discrepancies, the 76 samples misclassified by *Gel-Fn-PCR* were re-analysed by the real-time PCR (*F-RT-PCR*) published by Ta-Tang et al. in 2022.^28^ Among the 62 samples classified as *L. loa*, 52 were confirmed, while 10 were reclassified as mixed infections, in agreement with microscopy. Among the 14 samples classified as *M. perstans*, seven were confirmed, while the remaining were reclassified as mixed infections (n = 4) or *L. loa* (n = 3). Overall, 77.6% (59/76) of discrepant results were concordant with *Gel-Fn-PCR*.

The *Gel-Fn-PCR* showed reduced concordance with microscopy for mixed infections. Several samples identified as mixed infections by microscopy were classified as single-species infections by *Gel-Fn-PCR*, suggesting an underestimation of co-infections by the molecular assay.

Additionally, 13 samples positive for *L. loa* by microscopy were negative by *Gel-Fn-PCR*, whereas 16 microscopy-negative samples were positive for *L. loa* by *Gel-Fn-PCR*; all were confirmed by F-RT-PCR (Table I). Unexpectedly, the sample #171 from Nigeria with 34550 *L. loa* mF/mL was missed by *Gel-Fn-PCR*, and doubtfully detected by *F-RT-PCR*.^28^

*Visualisation of amplified fragments* - Identification of the species was performed according to the size of the amplified fragments from the second PCR, as described in the original *Fn-PCR* method, which were 286 bp for *L. loa*, 301 bp for *W. bancrofti*, 305 bp for *M. ozzardi*, 312 bp for *M. perstans*, and 344 bp for *O. volvulus*.

**TABLE II:** Statistical analysis of the Gelified tube-filarial-nested polymerase chain reaction (Gel-Fn-PCR)

	*Loa loa*	*Mansonella perstans*	Mixed infections
Sensitivity	91.6% (87.8%, 95.4%)	95.0% (85.4%, 104.6%)	43.9% (35.6%, 52.1%)
Specificity	83.0% (79.6%, 86.4%)	97.7% (96.5%, 98.8%)	99.0% (98.2%, 99.9%)
PPV	70.3% (64.8%, 75.9%)	55.9% (39.2%, 72.6%)	92.4% (86.0%, 98.8%)
NPV	95.7% (93.7%, 97.7%)	99.8% (99.5%, 100.2%)	86.9% (84.2%, 89.6%)
Cohen's Kappa Index	0.688 (0.613, 0.762) Adequate	0.692 (0.619, 0.765) Adequate	0.532 (0.462, 0.601) Moderate

CI: confidence interval (95%); PPV: positive predictive value; NPV: negative predictive value.

*Statistical analysis* - The *Gel-Fn-PCR* showed good sensitivity and specificity values for *L. loa*, 91.6% and 83.0% respectively, and high sensitivity and specificity values for *M. perstans*, 95.0% and 97.7%, respectively. However, for mixed infections, sensitivity dropped to 43.9%, and specificity remained high at 99.0%. The positive predictive value (PPV) was low for both mono-infections, 70.3% for *L. loa* and 55.9% for *M. perstans*, but remained high for mixed infections at 92.4%. Negative predictive value (NPV) was high for all infection categories, 95.7% for *L. loa* infections, 99.8% for *M. perstans* infections*,* and 86.9% for mixed infections. Cohen's Kappa coefficient was between adequate and moderate. The detailed results of the statistical analysis are presented in Table II.

## DISCUSSION

In a landscape in which many endemic countries are close to eliminating human filariasis, the demand for reliable diagnostic methods is more critical than ever.^29^ ^,^ ^30^ ^,^ ^31^ Nucleic acid-based assays, which directly target parasite DNA, offer higher sensitivity and specificity than traditionally used immunoenzymatic methods.^32^ Therefore, this study developed the *Gel-Fn-PCR*, a reliable nucleic acid-based diagnostic method to detect all human filariasis while being more compatible with the POCTs criteria.

Considering microscopic diagnosis as the reference method, the *Gel-Fn-PCR* was able to identify most mono-infections with minimal discrepancies. Thirteen *L. loa* microscopy-positives were missed by *Gel-Fn-PCR* and, interestingly, all of them came from Nigeria. Twelve of those samples had a microfilariae load ranged 100-4150 mF/mL. The lower detection of microfilariae in some samples from Nigeria may be explained by the use of venous blood to prepare DBS. If blood samples were not fully homogenised prior to spotting, microfilariae may have been unevenly distributed, leading to potential loss of parasites in the analysed spots and consequently affecting detection sensitivity. The failure to detect sample #171, despite its high microfilaremia, may be related to pre-analytical factors associated with sample preparation, incomplete homogenisation prior to spotting may lead to the absence of parasite DNA in the analysed punch. This highlights a potential limitation of DBS-based approaches, where parasite distribution within the sample may not be homogeneous. Because the result was uncertain, two *L. loa*-specific methods were also performed specifically for this sample: the nested PCR published by Touré et al.^33^ and the LAMP published by Drame et al.^34^ Surprisingly, both methods yielded negative results for sample #171. Suspecting that it could be a *L. loa*-like parasite, the nematode-specific PCR developed by Casiraghi et al. was carried out.^35^ This nematode-specific PCR resulted in good amplification, and the sequencing of this fragment showed highest similarity with the species *L. loa*. To this day, the reason why discrepant results were obtained with different tests remains unclear. Since the available amount of blood was completely used during this study, and the microscopy slides were destroyed when diagnosis had been finished, further investigation was not possible.

Most discrepancies occurred with mixed infection samples. Microscopy was able to detect more mixed infections than the *Gel-Fn-PCR*, which detected most of these mixed infections as either single *L. loa* or *M. perstans* infections. *L. loa* microfilariae were usually found in greater proportion than *M. perstans* microfilariae in most mixed infections, and since *Gel-Fn-PCR* included a single forward and a single reverse primer targeting all filarial species equally, amplification was likely biased towards the more abundant species. This was the reason why the mixed infection detected by microscopy was identified mostly as *L. loa* by *Gel-Fn-PCR*.

On the other hand, eighteen samples that were negative by microscopy were positive by *Gel-Fn-PCR*. These results were confirmed by *F-RT-PCR*.^28^ Most of these discordant results derived again from Nigerian samples, whose microscopic diagnosis was performed employing thin blood smears. Thin blood smears require only from 5 to 10 µL of blood, and it has less sensitivity than thick blood smears, which require up to 30 µL.^36^ If Nigerian microscopists had used thick blood smears instead of thin blood smears, perhaps there wouldn't have been many discordant results between microscopy and *Gel-Fn-PCR*.

According to statistical analysis, the *Gel-Fn-PCR* demonstrated high sensitivity and specificity for the detection of mono-infections with 95% CI indicating consistent performance. These results support the reliability of the assay for diagnosing single-species filarial infections when compared with microscopy as the reference standard.

Regarding mixed infections, *Gel-Fn-PCR* was not a reliable method for diagnosing them. PPV was, as expected, comparatively lower for both mono-infections than for mixed infections, since they were influenced by the numerous mixed infections that were misdiagnosed as mono-infections, which likely confounded the apparent diagnostic performance of the *Gel-Fn-PCR* for mono-infections and prevented a clear interpretation of its true diagnostic capacity. In contrast, the NPV remained high for all types of infections, thereby highlighting the robust reliability of the assay in accurately identifying negatives.

Cohen's kappa index indicated that the *Gel-Fn-PCR* had an adequate correlation with the microscopic diagnosis for mono-infections, while for mixed infections, the correlation was moderate, thereby corroborating our findings, and indicating that the *Gel-Fn-PCR* is a reliable diagnostic method for mono-infections, consistent with microscopy, although it is comparatively less consistent in the detection of mixed infections.

Various multiplex PCRs have also been developed and validated for the diagnosis of other parasites, including *Plasmodium* spp., *Schistosoma* spp., and soil-transmitted helminths. Still, the diagnosis of mixed infections remains a challenge for many researchers.^37^-^42^ This limitation is intrinsic to the multiplex PCR technique. Preferential amplification of one target over the others can be attributable to the inherent properties of the target DNA or to the fluctuation in the interactions of PCR reagents, especially when there is a low concentration of the target DNA.^43^

*Gel-Fn-PCR* offers advantages over other filarial PCR assays previously described in the literature. The PCR developed by Casiraghi et al.^35^ had high sensitivity and specificity, but species identification requires sequencing, thereby making it inapplicable as a POCT. In 2005, Nuchprayoon et al. developed a high sensitive and specific pan-filaria PCR, however, the method needs an additional restriction enzyme digestion step prior to the visualisation of the amplified fragments for species identification.^44^ This additional step increases the cost, duration, and technical complexity of the assay, further limiting its feasibility for implementation as a POCT.

Several multiplex PCR assays targeting filarial parasites have also been reported. Nicolas and Scoles developed in 1997 a multiplex PCR capable of detecting *Dirofilaria immitis* and *W. bancrofti*, although its application was limited to vector samples.^45^ Dyab et al.^46^ also published a multiplex PCR that detected *D. immitis*, *W. bancrofti*, and *D. repens*, yet this assay had only been evaluated in vectors.^46^ Finally, Mishra et al. proposed another multiplex PCR restricted to the detection of *B. malayi* and *W. bancrofti*, which was tested in both blood and vector samples.^47^ The *Gel-Fn-PCR* demonstrates a notably broader diagnostic spectrum compared with all the aforementioned methods, allowing the detection of all eight filarial species known to infect humans within a single assay.^45^ ^,^ ^46^ ^,^ ^47^

The *Gel-Fn-PCR* developed in this study has the same advantages of the *Fn-PCR* in terms of versatility, but with the addition of gelification technology benefits, such as convenient storage at 4ºC, shipment conditions, minimal set-up time, fewer errors, as well as being more user-friendly. Furthermore, *Gel-Fn-PCR* is cost-effective, 0.78€ per reaction. Although the *Gel-Fn-PCR* is still a relatively complex procedure and requires specialised equipment, and therefore cannot yet be considered a true POCT, the findings of this study demonstrate that PCR-based methods are approaching practical feasibility as POCTs in the diagnosis of human filariasis.

*In conclusion* - The *Gel-Fn-PCR* represents an optimised adaptation of the previously developed conventional *Fn-PCR,* maintaining comparable sensitivity and specificity for the detection of single filarial infections while offering important operational advantages. In particular, the gelified format simplifies the nested PCR workflow by reducing reagent preparation and handling steps, improving assay standardisation, and lowering the risk of contamination associated with multi-step procedures. These features make the *Gel-Fn-PCR* more practical and cost-effective for routine laboratory use. Sensitivity for mixed infections was low, though specificity remained high. While the assay was suboptimal for detecting mixed infections, particularly when one species was predominant, it performed effectively for single-species detection. Overall, the *Gel-Fn-PCR* is particularly advantageous in laboratory settings requiring simplified workflows and standardised procedures, while retaining the diagnostic performance of the original *Fn-PCR*. The *Gel-Fn-PCR* demonstrated limitations as a deployable POCT in resource-limited areas; nonetheless, this study highlighted the feasibility of integrating gelification technology with nucleic acid-based assays. It underscores the potential of using gelified tubes as a platform for further development of multiplexed and field-adaptable molecular tools.

*Study limitations* - A limitation of this study was the comparison between *Gel-Fn-PCR* and the original *Fn-PCR* only performed on a limited subset of samples during the optimisation phase, rather than across the entire dataset. Given that the *Gel-Fn-PCR* has only been tested on *L. loa* and *M. perstans* positive samples, further validation with additional blood-borne filarial species will be essential to confirm its broad diagnostic utility. Similarly, *Gel-Fn-PCR* should be tested on skin snip samples to determine whether this method would be valid for detecting *O. volvulus* microfilariae. Moreover, the Nigerian samples had been stored for a relatively long period of time, approximately ten years, and DBS samples have been prepared from venous blood, which may have affected the test results.

## Data Availability

After publication the data will be available on demand to corresponding author.

## References

[1] WHO - World Health Organization (2021). Ending the neglect to attain the Sustainable Development Goals: a road map for neglected tropical diseases 2021-2030. https://www.who.int/publications/i/item/9789240010352.

[2] Rahmah N, Taniawati S, Shenoy RK, Lim BH, Kumaraswami V, Anuar AK (2001). Specificity and sensitivity of a rapid dipstick test (Brugia Rapid) in the detection of Brugia malayi infection. Trans R Soc Trop Med Hyg.

[3] Nikièma AS, Koala L, Unnasch TR, Diendéré J, Compaoré J, Ouédraogo MW (2024). Field sensitivity and specificity of the SD BIOLINE onchocerciasis IgG4 Rapid Diagnostic Test in children < 10 years old from endemic areas in Burkina Faso. Parasit Epidemiol Control.

[4] Weil GJ, Curtis KC, Fakoli L, Fischer K, Gankpala L, Lammie PJ (2013). Laboratory and field evaluation of a new rapid test for detecting *Wuchereria bancrofti* antigen in human blood. Am Soc Trop Med Hyg.

[5] Graves PM, Scott JL, Berg Soto A, Widi AYN, Whittaker M, Lau CL (2025). Laboratory comparison of rapid antigen diagnostic tests for lymphatic filariasis: STANDARD Q Filariasis Antigen Test (QFAT) versus Bioline Filariasis Test Strip (FTS). Trop Med Infect Dis.

[6] Scott JL, Mayfield HJ, Sinclair JE, Martin BM, Howlett M, Muttucumaru R (2024). Field laboratory comparison of STANDARD Q Filariasis Antigen Test (QFAT) with Bioline Filariasis Test Strip (FTS) for the detection of Lymphatic Filariasis in Samoa, 2023. PLoS Negl Trop Dis.

[7] Pietrzak D, Łuczak JW, Wiśniewski M (2024). Beyond tradition: exploring cutting-edge approaches for accurate diagnosis of human filariasis. Pathogens.

[8] Gbakima AA, Nutman TB, Bradley JE, McReynolds LA, Winget MD, Hong Y (1996). Immunoglobulin G subclass responses of children during infection with *Onchocerca volvulus*. Clin Diagn Lab Immunol.

[9] Bharadwaj M, Bengtson M, Golverdingen M, Waling L, Dekker C (2021). Diagnosing point-of-care diagnostics for neglected tropical diseases. PLoS Negl Trop Dis.

[10] Hertz MI, Nana-Djeunga H, Kamgno J, Njouendou AJ, Chunda VC, Wanji S (2018). Identification and characterization of *Loa loa* antigens responsible for cross-reactivity with rapid diagnostic tests for lymphatic filariasis. PLoS Negl Trop Dis.

[11] Alhassan A, Li Z, Poole CB, Carlow CKS (2015). Expanding the MDx toolbox for filarial diagnosis and surveillance. Trends Parasitol.

[12] Tang T-HT, López-Vélez R, Lanza M, Shelley AJ, Rubio JM, Luz SLB (2010). Nested PCR to detect and distinguish the sympatric filarial species *Onchocerca volvulus*, *Mansonella ozzardi* and *Mansonella perstans* in the Amazon Region. Mem Inst Oswaldo Cruz.

[13] Madejon Seiz A, Limones Lopez G, Haro Castuera A, De Grado Sanz M, Franco de Sarabia Rosado PM (2003). Stabilised composition for fluorimetric, colorimetric or chemoluminescent assay, kits containing same and production method thereof. Espanha patent.

[14] Sun Y, Hogberg J, Christine T, Florian L, Monsalve LG, Rodriguez S (2013). Pre-storage of gelified reagents in a lab-on-a-foil system for rapid nucleic acid analysis. Lab Chip.

[15] Lahane V, Sagar VVSS, Naik S, Shukla S, Acharya S, Kumar S (2022). Microfilaria causing nephrotic syndrome: Highlighting the importance of diethylcarbamazine provocation test for diagnosis. J Fam Med Prim Care.

[16] Rajamanickam A, Babu S (2025). Unraveling the dynamics of human filarial infections: immunological responses, host manifestations, and pathogen biology. Pathogens.

[17] Dickson B, Graves P, McBride W (2017). Lymphatic filariasis in mainland southeast Asia: a systematic review and meta-analysis of prevalence and disease burden. Trop Med Infect Dis.

[18] Chavarkar SP (2016). Lymphatic filariasis: the importance of screening all peripheral blood smears in low power for detection of asymptomatic cases. Int J Res Med Sci.

[19] Medeiros ZM, Vieira AVB, Xavier AT, Bezerra GSN, Lopes MDFC, Bonfim CV (2021). Lymphatic filariasis: a systematic review on morbidity and its repercussions in countries in the Americas. Int J Environ Res Public Health.

[20] Ton TGN, Mackenzie C, Molyneux DH (2015). The burden of mental health in lymphatic filariasis. Infect Dis Poverty.

[21] WHO - World Health Organization / Region African ESPEN Annual Report 2024. Expanded special project for elimination of neglected tropical diseases. https://espen.afro.who.int/tools-resources/documents/espen-annual-report-2024.

[22] Capote-Morales R, Benito A, Berzosa P, de la Fuente IM, Akindele AA, Cruces R (2025). A novel filarial-multiplexed probe-quantitative PCR for the advance in the diagnosis of multiple infections with human filariasis. Trop Med Int Health.

[23] Malsagova K, Kopylov A, Stepanov A, Butkova T, Izotov A, Kaysheva A (2020). Dried blood spot in laboratory: directions and prospects. Diagnostics.

[24] Demirev PA (2013). Dried blood spots: analysis and applications. Anal Chem.

[25] Sharma A, Jaiswal S, Shukla M, Lal J (2014). Dried blood spots: Concepts, present status, and future perspectives in bioanalysis. Drug Test Anal.

[26] WinEpi - Working in Epidemiology Reflexiones sobre el COVID-19 de un epidemiólogo veterinario. http://www.winepi.net/.

[27] Akobeng AK (2007). Understanding diagnostic tests 1: sensitivity, specificity and predictive values. Acta Paediatr.

[28] Ta-Tang TH, Febrer-Sendra B, Berzosa P, Rubio JM, Romay-Barja M, Ncogo P (2022). Comparison of three PCR-based methods to detect *Loa loa* and *Mansonella perstans* in long-term frozen storage dried blood spots. Trop Med Int Health.

[29] WHO - World Health Organization (2024). Global programme to eliminate lymphatic filariasis: progress report, 2023. https://www.who.int/publications/i/item/who-wer-9940-565-576.

[30] WHO - World Health Organization Elimination of human onchocerciasis: progress report, 2023-2024 = Élimination de l'onchocercose humaine : rapport de situation, 2023-2024. https://iris.who.int/items/0627ee12-87ef-4eab-b9b4-0f2db4e2d71e.

[31] Hietanen H, Pfavayi LT, Mutapi F (2025). Unlocking the blueprint to eliminating neglected tropical diseases: a review of efforts in 50 countries that have eliminated at least 1 NTD. PLoS Negl Trop Dis.

[32] Lubbers C, Amaral LJ, Colebunders R, Brattig N, Hadermann A (2025). The last mile in onchocerciasis elimination: diagnostic challenges. Trends Parasitol.

[33] Touré FS, Bain O, Nerrienet E, Millet P, Wahl G, Toure Y (1997). Detection of *Loa loa*-specific DNA in blood from occult-infected individuals. Exp Parasitol.

[34] Drame PM, Fink DL, Kamgno J, Herrick JA, Nutman TB (2014). Loop-mediated isothermal amplification for rapid and semiquantitative detection of *Loa loa* infection. J Clin Microbiol.

[35] Casiraghi M, Anderson TJC, Bandi C, Bazzocchi C, Genchi C (2001). A phylogenetic analysis of filarial nematodes: comparison with the phylogeny of *Wolbachia* endosymbionts. Parasitology.

[36] Eberhard ML, Lammie PJ (1991). Laboratory diagnosis of filariasis. Clin Lab Med.

[37] Ten Hove RJ, Verweij JJ, Vereecken K, Polman K, Dieye L, Van Lieshout L (2008). Multiplex real-time PCR for the detection and quantification of *Schistosoma mansoni* and *S. haematobium* infection in stool samples collected in northern Senegal. Trans R Soc Trop Med Hyg.

[38] Webster BL, Rollinson D, Stothard JR, Huyse T (2010). Rapid diagnostic multiplex PCR (RD-PCR) to discriminate *Schistosoma haematobium* and *S. bovis*. J Helminthol.

[39] Rubio JM, Benito A, Roche J, Berzosa PJ, García ML, Micó M (1999). Semi-nested, multiplex polymerase chain reaction for detection of human malaria parasites and evidence of *Plasmodium vivax* infection in Equatorial Guinea. Am J Trop Med Hyg.

[40] Kho WG, Chung JY, Sim EJ, Kim MY, Kim DW, Jongwutiwes S (2003). A multiplex polymerase chain reaction for a differential diagnosis of *Plasmodium falciparum* and *Plasmodium vivax*. Parasitol Int.

[41] Schols R, Carolus H, Hammoud C, Mulero S, Mudavanhu A, Huyse T (2019). A rapid diagnostic multiplex PCR approach for xenomonitoring of human and animal schistosomiasis in a 'One Health' context. Trans R Soc Trop Med Hyg.

[42] Phuphisut O, Yoonuan T, Sanguankiat S, Chaisiri K, Maipanich W, Pubampen S (2014). Triplex polymerase chain reaction assay for detection of major soil-transmitted helminths, *Ascaris lumbricoides*, *Trichuris trichiura*, *Necator americanus*, in fecal samples. Southeast Asian J Trop Med Public Health.

[43] Markoulatos P, Siafakas N, Moncany M (2002). Multiplex polymerase chain reaction: a practical approach. J Clin Lab Anal.

[44] Nuchprayoon S, Junpee A, Poovorawan Y, Scott AL (2005). Detection and differentiation of filarial parasites by universal primers and polymerase chain reaction-restriction fragment length polymorphism analysis. Am J Trop Med Hyg.

[45] Nicolas L, Scoles GA (1997). Multiplex polymerase chain reaction for detection of *Dirofilaria immitis* (Filariidea: Onchocercidae) and *Wuchereria bancrofti* (Filarioidea: Dipetalonematidae) in their common vector *Aedes polynesiensis* (Diptera: Culicidae). J Med Entomol.

[46] Dyab AK, Galal LA, Mahmoud AES, Mokhtar Y (2015). Xenomonitoring of different filarial nematodes using single and multiplex PCR in mosquitoes from Assiut Governorate, Egypt. Korean J Parasitol.

[47] Mishra K, Raj DK, Hazra RK, Dash AP, Supakar PC (2007). The development and evaluation of a single step multiplex PCR method for simultaneous detection of *Brugia malayi* and *Wuchereria bancrofti*. Mol Cell Probes.

