# Supplementary material for: Pan filarial-nested PCR with gelified tubes: a new approach towards point-of-care testing for human filariasis
Source: Mem Inst Oswaldo Cruz. 2026 Sep 25;121:e250337. doi: 10.1590/0074-02760250337 (PMC13623295; doi:10.1590/0074-02760250337)
Supplement: Peer review history

# Reviewer report: FIRST REVIEW ROUND - REVIEWERS' COMMENTS

## REVIEWER #1

While I can see that the presented article has been prepared by a team with a strong-track in developing molecular tools for filarial parasite detection, I am unconvinced the value of the novel Gel-Fn-PCR is adequately explained the current version of the authors 'manuscript. Thus, even though the article is written in good clear English, I feel there are a lot of presentational issues that prevent what has been done from being clearly understood. I do, however, trust that with some minor revisions the authors will be able to turn their submission into a valuable contribution to the field. Below, I have provided some suggestions as to how I think the article´s clarity could be improved by reconsidering how the results are presented and re-organizing the text. I have also highlighted specific passages of text that I think need to revised for accuracy and to improve clarity.

LINES 66-73: Here it is not made adequately clear that someone can test positive with one of the "antibody-based tests" they describe because they have been exposed to a given filarial parasite (e.g. o. volvulus), but may not have necessarily been infected with the parasite.

LINES 106-109: Please re-write this section to make clearer the benefits of the new Gel-Fn-PCR.

LINES 122-123: Please provide more information on how microfilariae counts were made. How much blood was examined to make these counts?

Line 127: this is not a good sub-title, please revise.

LINES 163: What is the sequence of the ITS1F-BIS primer? It is not provided in the cited reference. Also, why use a different primer from that described in Tang et al. (2010) if the claim in LINES 83-84 is true?

LINES 174-178: What were the positive controls used? What other filarial parasites might conceivably be detected in the study regions. What PCR fragment sizes would be generated from their DNA? Can you provide us with the gel results so that anyone wishing to employ the method can see how diagnostic calls get made?

LINES: 138-140 "is the requirement of basic and inexpensive" should surely be changed to something like "is the requirement of ONLY basic and inexpensive".

LINES 189: I recommend changing single infection (here and throughout) to single-species infections or mono-infections for clarity.

LINE 200: The subtitle needs changing; it is confusing. Are the blood samples placed on microscope slides not clinical samples?

LINES: 208-215: this belongs in the methods section.

LINES 219-221: While I think a table comparing the results of the two methodologies is useful, I feel the current matrix format is difficult to interpret. The current format makes it difficult to focus on the differences in diagnostic results observed between the microscopy and Gel-Fn-PCR approaches.

LINES 222-223: This needs to be we-written for clarity. As I understand it Gel-Fn-PCR routinely miss-reports co-infections as mono-infections. This should be made clearer, talking about detecting and not detecting makes the paper harder to follow. It would also be helpful to see gels from co-infection positive PCRs and PCR results where a co-infection is miss-reported by Gel-Fn-PCR as having a mon-infection.

LINES: 238-239: Please provide the PCR fragment sizes of all human filarial parasites known to cause blood parasitaemias in humans here.

LINES: 261-263: this belongs in the methods section

LINES: 264-265: this belongs in the results section.

LINES: 265-268: the belongs in the methods section

LINES: 268-272: Some of this sentence belongs in the methods section, some in the results section. None of it should appear in the discussion for the first time.

LINES: 287-288: This should be in the methods section.

LINES: 292-293: please clarify what statistical tests are being referred to and what definition of sufficiency is being used.

LINES: 328-331: this needs a reference or references.

LINES: 335-339: Is this really more cost-effective? I think a discussion of emergent microscope-based AI parasites identification tools is necessary here.

LINES: 341-349: The focus here needs to be on why Gel-Fn-PCR is better than conventional Fn-PCR assay this team previously developed. If it is only better in very specific situations, these situations need to be made clear. Most importantly, the authors need to discuss the automated QIAGEN electrophoresis system they use to diagnose species. How field friendly is it? Could it be replaced by an agarose gel electrophoresis system to make it more field-friendly?

LINES: 351-356: The Gel-Fn-PCR assay should have been compared to the standard Fn-PCR assay the team previously developed. This should be stated here. The differential handling of blood samples (which the authors discuss as having impacted their results) needs also to be mentioned here.

LINES: 560-565: This figure legend is less than helpful. Mentioning O. volvulus diagnosis and only O. volvulus here is very confusing. Please provide helpful information including information on the QIAGEN electrophoresis system they have used and show. More details on the QIAGEN electrophoresis system are also required in the methods section.

## AUTHORS' RESPONSE TO THE REVIEWERS

**Manuscript ID** MIOC-2025-0337 entitled "Pan filarial-Nested PCR with gelified tubes: a new approach towards point-of-care testing for human filariasis"

**Running headline:** "Gelified tubes for filaria molecular diagnosis"

Dear Dr. TA-TANG:

Manuscript ID MIOC-2025-0337 entitled "Pan filarial-Nested PCR with gelified tubes: a new approach towards point-of-care testing for human filariasis" Running headline: "Gelified tubes for filaria molecular diagnosis" which you submitted to the Memórias do Instituto Oswaldo Cruz, has been fully evaluated by independent peer reviewers and substantial concerns were raised about the manuscript as it currently stands. These issues must be addressed before we would be willing to consider a revised version of your study.

Please be aware that the submission of a new revised version of your manuscript does not guarantee eventual acceptance, being subject to re-review before a decision is rendered.

If you are able to revise your manuscript along the lines recommended by the referees within 90 days, a list of specific responses to each of the reviewer's comments, noting where you have made such changes in the revision, is needed.

We will address all Reviewer comments and send the revised version, along with a detailed point-by-point response, within the requested timeframe and, preferably, before the 90-day deadline so that the article can be published as soon as possible.

Authors are not mandated to agree with every suggestion made by the reviewer. However, reasons for contesting any reviewer comments must be provided. If those unresolved comments appear to be fundamental to the work being described, the revision may be resubmitted to the Editors for final decision.

We will carefully analyze all Reviewer comments and provide clear and consistent responses. Where necessary, we will include detailed explanations to clarify our reasoning and the revisions made to the manuscript.

The specific response to the reviewer's comments, the new version of the manuscript, including tables and legends to figures must be submitted. If it is the case, all figures (in TIFF format 300dpi), must be uploaded again separately with legends. Please upload the new version of manuscript in the "manuscript files" section as main documents.

To revise your manuscript, log into https://mc04.manuscriptcentral.com/mioc-scielo and enter your Author Center, where you will find your manuscript under «Manuscripts with Decisions.» Under «Actions,» click on «Create a Revision.» Your manuscript number has been appended to denote a revision.

You may also click the link below to start the revision process for your manuscript. If you use the link below you will not be required to login to ScholarOne Manuscripts.

*** PLEASE NOTE: This is a two-step process. After clicking on the link, you will be directed to a webpage to confirm. ***

https://mc04.manuscriptcentral.com/mioc-scielo?URL_MASK=c9b885fd528c4a819949a0396678c5c6

You will be unable to make your revisions on the originally submitted version of the manuscript. Instead, revise your manuscript using a word processing program and save it on your computer. Please also highlight the changes to your manuscript within the document by using the track changes mode in MS Word or by using bold or colored text.

Once the revised manuscript is ready, you can upload and submit through your Author Center.

When submitting your revised manuscript, you will be able to respond to the comments made by the reviewer(s) in the space provided. You can use this space to document any changes you make to the original manuscript. In order to expedite the processing of the revised manuscript, please be as specific as possible in your response to the reviewer(s).

IMPORTANT: Your original files are available to you when you upload your revised manuscript. Please delete any redundant files before completing the submission.

Once again, thank you for submitting your manuscript to the Memórias do Instituto Oswaldo Cruz and I look forward to receiving your revision.

Sincerely,

Dr. Adeilton Brandão

Handling Editor

Memórias do Instituto Oswaldo Cruz

## Reviewer 1

**LINES 66-73: Here it is not made adequately clear that someone can test positive with one of the "antibody-based tests" they describe because they have been exposed to a given filarial parasite (e.g. o. volvulus), but may not have necessarily been infected with the parasite.**

We thank the reviewer for this comment. We have clarified this point in the revised manuscript to better explain this limitation of serological assays. Antibody-based tests detect previous exposure and may not necessarily indicate active infection due to the persistence of circulating antibodies.

Revision in manuscript (Lines 69-72):

*Antibody-based tests detect the host immune response rather than the presence of the parasite itself. Positive serology does not necessarily indicate active infection, because circulating antibodies can persist for long periods after successful treatment or parasite clearance.*

**LINES 106-109: Please re-write this section to make clearer the benefits of the new Gel-Fn-PCR.**

We thank the reviewer for this suggestion. The objective of the study has been rewritten to better highlight the advantages of the *Gel-Fn-PCR* format. Specifically, we clarified that the gelified tube approach simplifies the nested PCR workflow by reducing reagent preparation and handling steps, facilitates standardization of the assay, lowers operational costs, and minimizes the risk of contamination, while maintaining the sensitivity and specificity of the original manual *Fn-PCR* method.

Revision in manuscript (Lines 109-118):

*Therefore, the objective of this study was to convert the original manual Fn-PCR into a gelified tube format, termed gelified tube-filarial-nested PCR (Gel-Fn-PCR), by applying a gelification process to the reaction components. This modification was intended to simplify the nested PCR workflow by reducing manual preparation steps and handling, thereby facilitating assay implementation in routine laboratory settings. In addition, the gelified format aims to improve standardization, reduce the risk of contamination associated with multiple reaction preparations, and lower operational costs. Finally, the Gel-Fn-PCR was optimized and validated for the laboratory diagnosis of human filariasis.*

**LINES 122-123: Please provide more information on how microfilariae counts were made. How much blood was examined to make these counts?**

We appreciate the reviewer's observation. Additional microscopy details have been included in the revised manuscript. The amount of blood used for microfilariae counting was approximately 20 µL and the entire thick blood smear was examined microscopically to count microfilariae. Microfilarial density was then expressed as microfilariae per milliliter of blood (mF/mL) based on the volume of blood examined.

Revision in manuscript (Lines 132-136):

*Each thick blood smear contained approximately 20 µL. The entire smear was systematically screened and the number of microfilariae was recorded. Microfilarial density was subsequently calculated and expressed as microfilariae per milliliter of blood (mF/mL) according to the volume of blood examined.*

**Line 127: this is not a good sub-title, please revise.**

We appreciate this helpful suggestion. The subtitle has been revised to better reflect the content of the section. It has been changed to "*2.2 Sample collection and preparation of dried blood spots (DBS)*" (Line 140).

**LINES 163: What is the sequence of the ITS1F-BIS primer? It is not provided in the cited reference. Also, why use a different primer from that described in Tang et al. (2010) if the claim in LINES 83-84 is true?**

We agree with the reviewer and have revised the manuscript accordingly. The sequence of the ITS1F-BIS primer has now been included in the revised manuscript. The primer sequence is 5′-GGTGAACCTGC**RGMW**GGATC-3′. This primer corresponds to a degenerate version of the original FIL-2F primer (GGTGAACCTGC**GGAA**GGATC). Degenerate bases were introduced to improve amplification of genetically diverse *Loa loa* isolates that were not efficiently amplified by the original primer sequence. Since the primer sequence was not explicitly reported in the previously cited reference, this citation has been removed and the primer information is now fully provided in the Materials and Methods section.

Revision in manuscript (Lines 176-180):

*In addition, the FIL2F primer (GGTGAACCTGCGGAAGGATC) from the second PCR was replaced by the ITS1F-BIS primer (5′-GGTGAACCTGCRGMWGGATC-3′), a degenerate version of the original FIL-2F primer, was used in this study to improve amplification of genetically diverse Loa loa isolates.*

**LINES 174-178: What were the positive controls used? What other filarial parasites might conceivably be detected in the study regions. What PCR fragment sizes would be generated from their DNA? Can you provide us with the gel results so that anyone wishing to employ the method can see how diagnostic calls get made?**

We thank the reviewer for these relevant questions.

Positive controls: Positive controls consisted of DBS samples previously diagnosed as positive for filarial infection by microscopy, as well as by other validated molecular diagnostic methods available in our laboratory.

What other filarial parasites might conceivably be detected in the study regions? In the samples analysed, only *Loa loa* and *Mansonella perstans* microfilariae were detected. These are among the filarial species known to circulate in the study regions. Other possible species that could potentially be detected in blood samples from Equatorial Guinea and Nigeria included *Wuchereria bancrofti*. Unfortunately (or fortunately for the people living in the region), no samples tested positive to *W. bancrofti*.

PCR fragment sizes: The *Gel-Fn-PCR* developed in this study is based on the previously described nested filarial PCR method (2010), and the gelified format does not modify the amplification targets or the expected amplicon sizes. Therefore, the fragment sizes generated correspond to those described in the original method: *O. volvulus* 344 bp, *M. perstans* 312 bp, *M. ozzardi* 305 bp, *W. bancrofti* 301 bp, *Loa loa* 286 bp.

Gel visualization: Below, an example of Qiaxcel multicapilar electrophoresis results to illustrate the diagnostic band patterns obtained with the Gel-Fn-PCR. *Note: this image has been taken from the poster presented at the 34^th^ ECCMID celebrated in Barcelona (Spain) 27-30 April 2024.

***Note:** this image has been taken from the poster presented at the 34^th^ ECCMID celebrated in Barcelona (Spain) 27-30 April 2024.

In our study, PCR products were analyzed using the QIAxcel (QIAGEN) automated electrophoresis system, which provides a standardized and time-efficient alternative to conventional agarose gel electrophoresis. This platform offers higher resolution and reproducibility in fragment size determination, reducing variability associated with manual gel preparation and interpretation.

Although the cartridge-based system may involve higher initial costs, its advantages in terms of reduced hands-on time, automation, and improved consistency can offset these limitations in laboratory settings with moderate to high sample throughput.

We would like to clarify that the use of the QIAxcel system is not essential for the application of the *Gel-Fn-PCR*. The assay can also be performed using conventional agarose gel electrophoresis, which remains a suitable and more accessible option for laboratories with limited resources. This clarification has been included in the revised manuscript.

Revision in manuscript (Lines 181-188):

*For each reaction performed, appropriate positive and negative controls were always included. Positive controls were DBS samples of L. loa and M. perstans diagnosed by microscopy, as well as by other validated molecular diagnostic methods available in our laboratory; negative controls were DBS samples of filarial microscopy-negative individuals from filarial endemic regions that were negative for filarial infection but could be positive or negative for other tropical parasites, such as Plasmodium* spp*. All samples were run in duplicate. If the duplicates varied (one positive, one negative), the samples were rerun in triplicate.*

(Lines 211-213)

*The Qiaxcel automated electrophoresis system allows rapid, standardized, and high-resolution fragment analysis. However, conventional agarose gel electrophoresis can also be used as an alternative, particularly in resource-limited settings.*

**LINES: 138-140 "is the requirement of basic and inexpensive" should surely be changed to something like "is the requirement of ONLY basic and inexpensive".**

Thanks for this correction. It has been changed in the revised manuscript (Line 151).

**LINES 189: I recommend changing single infection (here and throughout) to single-species infections or mono-infections for clarity.**

We think it is a good suggestion. In the revised manuscript "single infection" has been changed by "mono-infection" in the entire manuscript. Many thanks.

**LINE 200: The subtitle needs changing; it is confusing. Are the blood samples placed on microscope slides not clinical samples?**

Thanks for this objection. As in the M&M section, this subtitle has been changed to *3.2 Sample collection and preparation of dried blood spots (DBS)* in the revised manuscript (Line 238).

**LINES: 208-215: this belongs in the methods section.**

We respectfully disagree with the Reviewer's suggestion. We would like to clarify that this study focuses on the development and optimization of a modified PCR format. Therefore, the experiments evaluating different concentrations of primers, enzymes, amount of DNA templates, etc were part of the optimization process and presented as M&M. However, the final conditions how the *Gel-Fn-PCR* were established must be in Results section.

The M&M section describes the general procedures, while the Results section presents the outcomes of the optimization process that led to the final standardized protocol.

To improve clarity, the section subtitle has been changed and a sentence has been introduced in the revised manuscript to present the established assay conditions rather than methodological procedures.

Revision in manuscript (Lines 246-248):

*3.3.1 Final optimized conditions of the Gel-Fn-PCR assay*

*The following section describes the final optimized conditions of the Gel-Fn-PCR assay established after systematic evaluation of multiple reaction parameters.*

**LINES 219-221: While I think a table comparing the results of the two methodologies is useful, I feel the current matrix format is difficult to interpret. The current format makes it difficult to focus on the differences in diagnostic results observed between the microscopy and Gel-Fn-PCR approaches.**

We thank the reviewer for this valuable comment because it has helped us to improve the manuscript. We agree that, while the full matrix provides comprehensive information, it may be difficult to quickly identify concordant and discordant results between methods.

To improve clarity, we have revised the presentation of the results by highlighting concordant results in the Table 1. This allows clearer visualization of the agreement and discrepancies between microscopy and *Gel-Fn-PCR*.

In the revised manuscript:

**Table 1:** This allows clearer visualization of the agreement and discrepancies between microscopy and *Gel-Fn-PCR*

In addition, a brief explanatory paragraph has been included in the Results section to guide the reader in interpreting the comparison between both methods.

Revision in manuscript (261-263)

*Overall agreement between microscopy and Gel-Fn-PCR was observed mainly along the diagonal (true concordant results), whereas discrepancies were observed in cases classified as mixed infections or negative by one of the methods.*

**LINES 222-223: This needs to be we-written for clarity. As I understand it Gel-Fn-PCR routinely miss-reports co-infections as mono-infections. This should be made clearer, talking about detecting and not detecting makes the paper harder to follow. It would also be helpful to see gels from co-infection positive PCRs and PCR results where a co-infection is miss-reported by Gel-Fn-PCR as having a mon-infection.**

We thank the Reviewer for this valuable comment. This validation section has been revised and almost entirely re-written to more clearly state that *Gel-Fn-PCR* tends to underestimate mixed infections, with some co-infections being classified as single-species infections.

Revision in manuscript (Lines 265-291)

*Microscopic diagnosis was able to detect more mixed infections, 21.03% (139/661), than the Gel-Fn-PCR, 9.99% (66/661). Most mixed infections missed by Gel-Fn-PCR (76/139) were classified as mono-infection, predominantly as L. loa (62/139) and to a lesser extent as M. perstans (14/139). To overcome these discrepancies, the 76 samples misclassified by Gel-Fn-PCR were re-analyzed by the real-time PCR (F-RT-PCR) published by Ta-Tang et al. in 2022.* ^28^ *Among the 62 samples classified as L. loa, 52 were confirmed, while 10 were reclassified as mixed infections, in agreement with microscopy. Among the 14 samples classified as M. perstans, 7 were confirmed, while the remaining were reclassified as mixed infections (n = 4) or L. loa (n = 3). Overall, 77.6% (59/76) of discrepant results were concordant with Gel-Fn-PCR.*

*The Gel-Fn-PCR showed reduced concordance with microscopy for mixed infections. Several samples identified as mixed infections by microscopy were classified as single-species infections by Gel-Fn-PCR, suggesting an underestimation of co-infections by the molecular assay.*

*Additionally, 13 samples positive for L. loa by microscopy were negative by Gel-Fn-PCR, whereas 16 microscopy-negative samples were positive for L. loa by Gel-Fn-PCR; all were confirmed by F-RT-PCR (Table 1).*

Regarding the gel to visualize co-infection positive PCRs and mono**-**infection PCRs, a representative electrophoresis image obtained using the QIAxcel system has been include below. This figure illustrates both correctly identified mixed infections and cases where mixed infections may be underrepresented and interpreted as single-species infections by the *Gel-Fn-PCR*.

**This figure:** illustrates both correctly identified mixed infections and cases where mixed infections may be underrepresented and interpreted as single-species infections by the *Gel-Fn-PCR*.

**LINES: 238-239: Please provide the PCR fragment sizes of all human filarial parasites known to cause blood parasitaemias in humans here.**

We thank the reviewer for this important comment. The expected PCR fragment sizes have now been included in the revised manuscript.

As the *Gel-Fn-PCR* is based on the previously described nested *Fn-PCR* method, the amplification targets and fragment sizes remain unchanged. Therefore, the fragment sizes correspond to those reported in the original method. Briefly, the expected fragment sizes for the main human filarial species are: *O. volvulus 344 bp, M. perstans 312 bp, M. ozzardi 305 bp, W. bancrofti 301 bp, Loa loa 286 bp.*

This information has now been explicitly added to the manuscript, with reference to the original publication.

Revision in manuscript (Lines 295-298)

*Identification of the species was performed according to the size of the amplified fragments from the second PCR, as described in the original Fn-PCR method, which were 286 bp for L. loa, 301 bp for W. bancrofti, 305 bp for M. ozzardi, 312 bp for M. perstans, and 344 bp for O. volvulus.*

**LINES: 261-263: this belongs in the methods section**

We appreciate the Reviewer's perspective; however, we consider that this part should be in discussion section to explain the discrepancies in Nigerian samples. We have reformulated the phrase to explain the potential impact of incomplete homogenization in the distribution of microfilariae that can affect the detection of the method.

Revision in Manuscript (Lines 323-327)

*The lower detection of microfilariae in some samples from Nigeria may be explained by the use of venous blood to prepare DBS. If blood samples were not fully homogenized prior to spotting, microfilariae may have been unevenly distributed, leading to potential loss of parasites in the analyzed spots and consequently affecting detection sensitivity.*

**LINES: 264-265: this belongs in the results section.**

We agree with the Reviewer and have revised the manuscript accordingly. The description of sample #171 has been revised to separate the objective observation from its interpretation. The result (failure of detection despite high microfilaremia) has been included in the Results section (Lines 292-293), while the potential explanation has been changed in the Discussion section.

Revision in Manuscript (Lines 292-293)

*Unexpectedly, the sample #171 from Nigeria with 34550 L. loa mF/mL was missed by Gel-Fn-PCR, and doubtfully detected by F-RT-PCR*.^28^

Revision in Manuscript (Lines 328-333)

*The failure to detect sample #171, despite its high microfilaremia, may be related to pre-analytical factors associated with sample preparation, incomplete homogenization prior to spotting may lead to the absence of parasite DNA in the analyzed punch. This highlights a potential limitation of DBS-based approaches, where parasite distribution within the sample may not be homogeneous.*

**LINES: 265-268: the belongs in the methods section**

We agree in part with the Reviewer and have modified the text to improve clarity. These additional PCR assays were not part of the standard methodology applied to all samples but were performed only for a single sample (#171) as confirmatory analyses due to an unexpected result. Therefore, we have retained their description in the Discusion section to reflect the case-specific investigation.

However, to improve clarity, we have added a brief statement in Materials and Methods section indicating that additional confirmatory molecular assays may be performed in selected cases when required.

Revision in manuscript (Lines 203-204)

*In selected cases with inconclusive or unexpected results, additional confirmatory molecular assays were performed using previously published methods.*

**LINES: 268-272: Some of this sentence belongs in the methods section, some in the results section. None of it should appear in the discussion for the first time.**

While we understand the Reviewer's point, we have retained this information in the Discussion section, as these additional analyses were performed exclusively for a single sample (#171) in order to investigate and interpret an unexpected result. Our intention in this section is to discuss the observed discrepancy and the steps taken to resolve it, rather than to describe standard methodological procedures applied across the study.

A general statement indicating that additional molecular methods may be used in selected cases has already been included in the Materials and Methods section. Therefore, we consider that the current structure appropriately distinguishes between general methodology and case-specific interpretation.

**LINES: 287-288: This should be in the methods section.**

We thank the reviewer for this comment, but we have maintained this information in the Discussion section, as it was intended to interpret the observed discrepancies between microscopy and *Gel-Fn-PCR* results rather than to describe the methodology itself.

Specifically, this statement provides a possible explanation for the discordant findings by highlighting how differences in microscopy techniques (thin versus thick blood smears) may influence diagnostic sensitivity. The general description of the microscopy methods has already been included in the Materials and Methods section. In addition, the volumes of blood used for thick and thin blood smears have now been explicitly specified in the Materials and Methods section to improve clarity (Line 129 for thin smears; Line 133 for thick smears). Therefore, we consider that its discussion in this context is appropriate.

**LINES: 292-293: please clarify what statistical tests are being referred to and what definition of sufficiency is being used.**

We agree with the Reviewer that this sentence is confusing. This sentence has been revised to explicitly refer to the statistical parameters calculated (sensitivity and specificity) and to avoid the use of the term "sufficient," which may be ambiguous. We have also incorporated reference to the 95% confidence intervals to better support the robustness of the results.

Revision in manuscript (Lines 359-363)

*According to statistical analysis, the Gel-Fn-PCR demonstrated high sensitivity and specificity for the detection of mono-infections with 95% CI indicating consistent performance. These results support the reliability of the assay for diagnosing single-species filarial infections when compared with microscopy as the reference standard.*

**LINES: 328-331: this needs a reference or references.**

Thank you for this note. We have now included appropriate references to support this statement (references 45--47 in the revised manuscript).

**LINES: 335-339: Is this really more cost-effective? I think a discussion of emergent microscope-based AI parasites identification tools is necessary here.**

We respectfully disagree with the Reviewer's suggestion. On one hand, the cost-effectiveness of the *Gel-Fn-PCR* primarily reduce reagent handling, simplified workflow, and operational efficiency compared to the conventional Fn-PCR.

On the other hand, emerging AI-based microscopy tools, while we acknowledge their relevance in the field of parasitological diagnosis, these approaches fall outside the scope of the present study, which is focused on the development and optimization of a molecular diagnostic method. Therefore, we have not included a detailed discussion of these technologies in discussion section.

**LINES: 341-349: The focus here needs to be on why Gel-Fn-PCR is better than conventional Fn-PCR assay this team previously developed. If it is only better in very specific situations, these situations need to be made clear. Most importantly, the authors need to discuss the automated QIAGEN electrophoresis system they use to diagnose species. How field friendly is it? Could it be replaced by an agarose gel electrophoresis system to make it more field-friendly?**

We thank the reviewer for this important comment. The Conclusions section has been revised to more clearly highlight the advantages of the *Gel-Fn-PCR* compared to the original *Fn-PCR* assay. Specifically, we now emphasize that the gelified format simplifies the nested PCR workflow by reducing reagent preparation and handling steps, improves standardization, and decreases the risk of contamination, while maintaining comparable diagnostic performance for single infections.

We have also clarified that the advantages of the *Gel-Fn-PCR* are particularly relevant for routine laboratory settings, whereas its lower sensitivity for mixed infections may limit its use in specific epidemiological contexts.

Regarding electrophoresis, we have clarified that although an automated QIAxcel (QIAGEN) system was used in this study to standardize fragment analysis, it is not required for the application of the method. The *Gel-Fn-PCR* can be performed using conventional agarose gel electrophoresis, making it adaptable to laboratories with more limited resources. This point has been explicitly included in both the Methods and Discussion sections.

Revision in manuscript (413-429)

*The Gel-Fn-PCR represents an optimized adaptation of the previously developed conventional Fn-PCR, maintaining comparable sensitivity and specificity for the detection of single filarial infections while offering important operational advantages. In particular, the gelified format simplifies the nested PCR workflow by reducing reagent preparation and handling steps, improving assay standardization, and lowering the risk of contamination associated with multi-step procedures. These features make the Gel-Fn-PCR more practical and cost-effective for routine laboratory use. Sensitivity for mixed infections was low, though specificity remained high. While the assay was suboptimal for detecting mixed infections, particularly when one species was predominant, it performed effectively for single-species detection. Overall, the Gel-Fn-PCR is particularly advantageous in laboratory settings requiring simplified workflows and standardized procedures, while retaining the diagnostic performance of the original Fn-PCR. The Gel-Fn-PCR demonstrated limitations as a deployable POCT in resource-limited areas; nonetheless, this study highlighted the feasibility of integrating gelification technology with nucleic acid-based assays. It underscores the potential of using gelified tubes as a platform for further development of multiplexed and field-adaptable molecular tools.*

**LINES: 351-356: The Gel-Fn-PCR assay should have been compared to the standard Fn-PCR assay the team previously developed. This should be stated here. The differential handling of blood samples (which the authors discuss as having impacted their results) needs also to be mentioned here.**

We thank the reviewer for this important comment. A direct comparison between the *Gel-Fn-PCR* and the original *Fn-PCR* assay was performed during the initial phase of the study as part of the optimization process. A subset of samples was analyzed in parallel using both methods to confirm that the gelified format yielded comparable results to the standard *Fn-PCR*.

Once equivalence between both approaches was established, subsequent analyses were carried out using the *Gel-Fn-PCR* assay. For the evaluation of diagnostic performance in a larger sample set, microscopy was used as the reference method, as it represents the routine diagnostic standard in the study settings.

We agree that this point was not sufficiently explained in the original manuscript. The text has been revised to clarify that an initial parallel comparison was performed and to explain the rationale for using microscopy as the reference method in the full dataset.

Revision in manuscript (Lines 191-196)

*In the course of the initial optimization phase, a subset of samples was analyzed in parallel using both the original Fn-PCR and the Gel-Fn-PCR to confirm the equivalence of the two approaches. Once comparable performance was established, subsequent analyses were performed using only the Gel-Fn-PCR assay. For the evaluation of diagnostic performance in the full dataset, microscopy was used as the reference method, as it represents the routine diagnostic standard in the study settings.*

Furthermore, a new limitation has now been included in the revised manuscript in *6. Study limitations* (Lines 434-436)

*A limitation of this study was the comparison between Gel-Fn-PCR and the original Fn-PCR only performed on a limited subset of samples during the optimization phase, rather than across the entire dataset.*

LINES: 560-565: This figure legend is less than helpful. Mentioning O. volvulus diagnosis and only O. volvulus here is very confusing. Please provide helpful information including information on the QIAGEN electrophoresis system they have used and show. More details on the QIAGEN electrophoresis system are also required in the methods section.

We thank the reviewer for this comment and agree that the figure legend was not sufficiently clear.

The legend has been revised to clarify that the asterisks refer to differences in the workflow depending on the type of sample analyzed. Specifically, samples obtained from skin snips (for the detection of *Onchocerca volvulus*) follow a different processing workflow compared to blood samples used for the detection of blood-dwelling filarial parasites. This distinction has been explicitly clarified by changing the legend of Figure 1 to avoid confusion.

Revision in manuscript (Lines 643-647)

Workflow of the Gel-Fn-PCR assay for the detection of filarial parasites. Asterisks (*) indicate steps that differ when the type of clinical sample are skin snip samples used for the detection of Onchocerca volvulus, a modified workflow is applied compared to the procedure used for blood samples targeting blood-dwelling filarial species (e.g., Wuchereria bancrofti, Loa loa and Mansonella perstans).

In addition, further details on the QIAGEN electrophoresis system have been included in the Methods section to improve reproducibility and clarity.

# Reviewer report: REVIEWERS' COMMENTS

## REVIEWER #1

I am now satisfied that the authors have addressed all the concerns I raised in my first review of their work. I look forward to seeing their work published in the near future and wish them well!
